# Cell-Penetrating Peptides: Design Strategies beyond Primary Structure and Amphipathicity

**DOI:** 10.3390/molecules22111929

**Published:** 2017-11-08

**Authors:** Daniela Kalafatovic, Ernest Giralt

**Affiliations:** 1 Institute for Research in Biomedicine (IRB Barcelona), The Barcelona Institute of Science and Technology (BIST), Baldiri Reixac, 10, 08028 Barcelona, Spain; daniela.kalafatovic@irbbarcelona.org; 2 Department of Inorganic and Organic Chemistry, University of Barcelona, Marti i Franques, 1-5, 08028 Barcelona, Spain

**Keywords:** cell-penetrating peptides, design, folding, rigidity, self-assembly

## Abstract

Efficient intracellular drug delivery and target specificity are often hampered by the presence of biological barriers. Thus, compounds that efficiently cross cell membranes are the key to improving the therapeutic value and on-target specificity of non-permeable drugs. The discovery of cell-penetrating peptides (CPPs) and the early design approaches through mimicking the natural penetration domains used by viruses have led to greater efficiency of intracellular delivery. Following these nature-inspired examples, a number of rationally designed CPPs has been developed. In this review, a variety of CPP designs will be described, including linear and flexible, positively charged and often amphipathic CPPs, and more rigid versions comprising cyclic, stapled, or dimeric and/or multivalent, self-assembled peptides or peptido-mimetics. The application of distinct design strategies to known physico-chemical properties of CPPs offers the opportunity to improve their penetration efficiency and/or internalization kinetics. This led to increased design complexity of new CPPs that does not always result in greater CPP activity. Therefore, the transition of CPPs to a clinical setting remains a challenge also due to the concomitant involvement of various internalization routes and heterogeneity of cells used in the in vitro studies.

## 1. Introduction

Cell-penetrating peptides (CPPs) are short peptides (<30 amino acids long) that are able to penetrate biological membranes and drive the internalization of a bioactive cargo in cells. An alternative terminology, the protein transduction domain (PTD), was used initially to describe such peptides as the first examples of CPPs were based on natural peptides derived from protein fragments [1]. Discovered in the 1990s, the first reported examples of CPPs were the Tat peptide (positions 48–60, derived from the transcription protein of HIV-1) [2,3] and penetratin (derived from the amphiphilic Drosophila Antennapedia homeodomain) [4]. Inspired by these molecules and focused mainly on positively charged sequences, many CPPs with differences in charge, polarity and/or structure have been discovered and/or designed [5,6]. Positively and negatively charged, amphipathic (primary or secondary) and non-amphipathic CPPs are used today in in vitro and in vivo studies [1,7,8,9,10].

**Figure 1:**
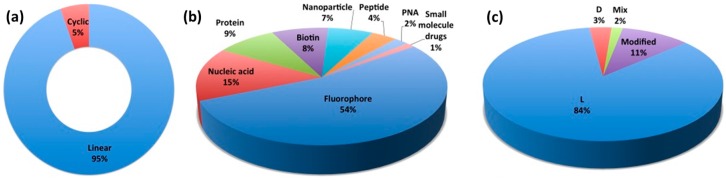
Statistical graphic representations depicting the distribution of cell-penetrating peptides (CPPs) reported in the literature (based on 1855 entries, of which 1699 are unique peptides) on the basis of the following: (**a**) linear and cyclic structure; (**b**) type of cargo delivered; and (**c**) chirality (taken from the CPP 2.0 database), taken from reference [12].

A CPP database, established in 2012 and now updated to CPP version 2.0, is a useful source of information [11,12]. Interestingly, the statistics section of the CPP database site (Figure 1) reveals that there are 1699 unique CPP sequences and that most are linear CPPs (94.5%) (Figure 1a) used for the delivery of fluorophores (58.4%) (Figure 1b). The major cargo of biomedical relevance is nucleic acids (16%). In addition, proteins (9%), nanoparticles (7.8%) and peptide therapeutics (4.7%) are other cargoes of interest (Figure 1b). The main body of research on CPPs focuses on synthetic peptides (54.8%) based on L-amino acids (84.3%) (Figure 1c). Moreover, the internalization efficiency and stability of all D-stereoisomers have been reported alongside CPP sequences composed of L- and D- combinations (Figure 1c) [11,12].

Extensive literature is available on the structure–activity relationship of primary CPPs and the folding properties of amphipathic peptides [1,7,12,13]. Charge and amphipathicity are the two main structural parameters taken into consideration when designing CPPs. Moreover, internalization can vary in function of overall amphipathicity and polycationic nature. In addition, the role of properties such as sequence length and conformation in internalization is equally as important as the accumulation of positive charge [7]. 

The folding ability of CPPs to form α-helices and β-sheets increases their efficiency to penetrate cells, as described in more detail in Section 4.2 [10,14,15]. However, the challenge is to introduce this property in a controlled and programmable manner through rational design. This review will address new concepts and strategies through which the rational design of CPPs can serve to enhance the efficiency of internalization and regulate the kinetics of this process.

## 2. From Protein Domains to the Design of Peptides with Cell Penetration Properties

### 2.1. Natural and Fusion Sequences

Many key parameters, such as origin [16], translocation mechanisms [7], physico-chemical properties [9] and others [17], have been proposed for the classification of CPPs. According to Eiríksdóttir et al., CPPs can be placed into the following three main groups: PTDs (Tat, Penetratin, etc.); model peptides (R9, KLAK); and designed peptides (Pep-1, sequence: KETWWETWWTEWSQPKKKRKV) [15]. Inspired by Tat (GRKKRRQRRRPQ) and penetratin (RQIKIWFQNRRMKWKK), the design of model CPPs is often based on small, cationic peptides (with charge ~5+) with low sequence diversity, such as polyarginines and polylysines. The most widely studied oligoarginine-based CPPs are R_8_ and R_9_ [18]. The uptake efficiency of polycationic CPPs was shown to depend on sequence length and also on the position of the arginine residue in the peptide sequence [19,20]. Recently, cyclization has been reported to maximize arginine contacts with the membrane, thereby achieving higher uptake efficiencies [21]. 

The first designed CPPs sought to increase the efficiency of individual known CPPs by including fusion sequences containing specific functions of interest [15]. An example is Pep-1, which includes the following: a hydrophobic tryptophan-rich segment (five tryptophan residues) to favor hydrophobic interactions with membranes; a positively charged lysine-rich segment (KKKRKV), used as a nuclear localization sequence derived from a virus (SV-40 T-antigen); and a proline spacer to increase the flexibility of the sequence [22]. Similarly, MPG (GALFLGFLGAAGSTMGAWSQPKKKRKV) was designed to contain the same hydrophilic nuclear localization sequence added to a viral hydrophobic domain derived from the HIV-gp-41 segment [23]. The integration of cationic, hydrophobic and amphipathic segments in different combinations led to the discovery of more efficient CPPs [5].

Furthermore, Giralt and co-workers reported on the discovery of a different class of CPPs, namely proline-rich amphipathic peptides, such as SAP (sweet arrow peptide) and its derivatives [24]. The SAP sequence (VRLPPP)_3_ was derived from the N-terminus of γ-zein VHL(PPP)_8_, a storage protein of maize. This peptide was found to adopt a polyproline II helical structure and it has the ability to self-aggregate.

CPP design has improved in order to address various challenges such as internalization efficiency, endosomal escape efficiency, circulation times, and specificity and selectivity (for cells, tissues, diseases). An example to mention are cysteine rich peptides [25]. A novel CPP was derived from a toxin found in snake venom, crotamine, that contains two nuclear localization domains (crot(2–18) and crot(27–39)). A cysteine-rich decapeptide (CRWRWKCCKK) with enhanced internalization properties was identified through examination of various versions of crot(27–39) (sequence: KMDCRWRWKCCKK) by systematic substitution and/or deletion of amino acid residues, coupled to structure–activity relationship studies [25].

A recent example of hydrophobic viral peptide having cell penetrating properties is gH 625 (sequence: HGLASTLTRWAHYNALIRAF) [26,27]. This CPP, derived from Herpes Simplex virus type I, was used to improve the delivery of liposomes [28,29], quantum dots [30], dendrimers [31], intrinsically disordered proteins [32] and SPIONS [33]. 

### 2.2. Structural and Functional Plasticity

CPPs have interfacial activity [7]. For most, membrane binding requires electrostatic interactions and/or peptide amphipathicity. In the literature, two main families of CPPs, namely arginine-rich and proline-rich, have been widely described, as have their mechanisms of internalization [19,20,24,34]. Futaki and others examined the behavior of oligoarginines on the basis of their sequence length and position of arginine in the sequence [19,20]. Interestingly, the study of oligoarginines (R_n_) with n = 4–16 residues has shown that the optimal number of arginine residues for efficient translocation is n = 8 [20]. Giralt and co-workers reported on the polyproline CPPs that tend to assume the helical conformations responsible for their internalization [24].

Many other CPPs are amphipathic and are able to fold into α-helical and β-sheet-like structures [9]. These molecules are referred to as primary or secondary amphipathic CPPs, on the basis of their amphipathic primary sequence or the ability to assume amphipathicity when folded, respectively [9]. Some of them fold only in the presence of membranes while others tend to assume secondary structures in solution, before interaction with membranes [9]. In addition, these CPPs can undergo conformational changes upon contact with membranes [35]. 

This ability to assume a higher order of organization through folding (intramolecular weak interactions) can also be achieved via controlled self-assembly through intermolecular, non-covalent interactions between CPP monomeric units [36]. While folding is widely explored in the CPP field, little attention has been paid to the propensity of CPPs to self-assemble in a controlled fashion. Short peptide amphiphiles fold in a similar manner to that of proteins and their amino acid sequence confers self-assembly propensity [37]. Of note, α-helical and β-sheet-like assemblies are the most widely studied systems.

The examples of CPPs mentioned above reflect the enormous functional sequence space of these molecules. Sequence length and conformation are key parameters that drive internalization [15]. However, peptide sequence and structure (i.e., the primary and secondary organizational level, respectively) alone are not enough to define and/or predict the mechanism of action as internalization is also determined by factors such as peptide concentration, membrane lipid composition, cargo, charge, self-assembly state, folding properties of CPPs and their amphipathic character, response of the cell to the peptide, temperature, and ionic strength, among others [7].

Some important *challenges* regarding CPPs remain to be addressed, namely internalization/translocation efficiency, often through improved endosomal escape, lack of target specificity, stability to proteases, and cytotoxicity [38]. Although the first reports on CPPs were based on protein derivatives, rational design is now dominating research activity in the field. Synthetic tools have paved the way to explore new approaches to improve the cell penetration of CPPs and CPP-therapeutic conjugates, both covalent and non-covalent. In addition to combining various known peptide sequences and introducing specific amino acid residues (Arg, Lys, Trp, Cys) into CPP design to improve efficiency, hydrocarbon chains were included in these peptides in order to increase their circulation times [39]. Moreover, disease-specific targeting moieties such as RGD or homing peptide sequences were added to achieve cell and tissue targeting (e.g., cancer cells) [40,41]. A new direction comprises the development of activatable CPPs where a pH- or an enzyme-responsive moiety are added to the design [40,42,43]. This approach makes the peptides stimuli-responsive to the tumor microenvironment, a property that can result in increased selectivity [44,45]. In addition, cyclization and stapling were proposed to achieve increased metabolic stability but also higher internalization efficiency as a result of increased structural or conformational rigidity/stability. Alternatively, multivalency of covalent dimers (primary), stabilized helices (secondary/tertiary) and supramolecular structures (quaternary) can be used to improve internalization. In this review, we wish to highlight how chemistry and rational design contribute to the CPP field.

## 3. Mechanistic Challenges

### 3.1. Internalization Mechanisms

CPPs, with or without cargo, can enter cells actively (energy-dependent mechanism) or passively (energy-independent mechanism) [7]. The physical chemistry of peptide–membrane interactions is crucial for efficient cell penetration. Many factors, including high positive charge content, cell membrane composition, endosomal escape, cargo, amphipathicity and folding ability, influence the mechanism and efficiency of cell penetration [7], thus making internalization a complex process.

Initially, it was thought that CPPs entered cells through energy-independent mechanisms and mainly through direct translocation [46]. Later, it was found that these initial studies were biased by cell fixation artifacts and that various mechanisms might be involved simultaneously in cell entry of CPPs [46]. Since then, progress has been made in understanding the uptake mechanisms of CPPs, and it has been shown that endocytic mechanisms, and in particular micropinocytosis, are involved [19]. However, other endocytic pathways, namely clathrin- and caveolin-mediated endocytosis, also trigger the internalization of CPPs [24]. Futaki and co-workers found that macropinocytosis plays a crucial role in the cellular uptake of arginine-rich peptides [19]. However, these peptides can also be internalized by direct translocation through the plasma membrane [19]. Proline-rich CPPs, characterized by the presence of pyrrolidine rings, enter cells via caveolae- or lipid-raft-mediated endocytosis [24].

A comprehensive overview of the mechanisms of uptake of several CPPs on the basis of their physico-chemical properties has been given elsewhere [7]. Interestingly, Wimley and co-workers classified CPPs on the basis of their mechanism of internalization. According to those authors, CPPs can translocate by the following: (a) plasma membrane lysis; (b) spontaneous (passive) membrane translocation; (c) energy-dependent membrane translocation; (d) transient membrane disruption; and (e) energy-dependent membrane disruption. Membrane lysis is not a desired parameter when designing CPPs as it might result in cytotoxicity at low peptide concentrations [7]. A hallmark of CPPs is translocation without lysis or membrane disruption.

A better understanding of the CPP-internalization mechanisms allows improved rational design of selective and efficient CPPs. The main challenge continues to be an incomplete understanding of the precise mechanism of cell entry. It is well documented that several mechanisms are concomitantly involved in this process, and there is still active debate in the field about this topic. Importantly, for the variety of CPPs described to date, it is not possible to predict their behavior or internalization mechanism based on structural information alone. In the future, it would be advantageous to use specific design parameters to guide the desired internalization routes in a programmed manner. Progress has recently been made in this regard [21,47,48,49,50].

### 3.2. Membrane Composition

In addition to the sequence and structure of the peptides, another parameter that influences membrane interaction with CPPs and therefore CPP internalization efficiency is membrane composition [1,51]. Proteoglycans (heparin sulfate proteoglycans and syndecans)—components of the extracellular matrix and regulators of cell surface microdomains—are essential for interaction with CPPs through electrostatic binding. This electrostatic interaction facilitates the accumulation of CPPs at the cell surface and constitutes the first contact with the cell membrane. This first interaction activates a variety of internalization mechanisms that lead to CPP internalization. One of the major differences between CPPs is their ability to interact with the membrane/cellular surface components. For example, cationic peptides bind through electrostatic interactions and hydrogen bonding through the guanidinium group, therefore various endocytic pathways can act simultaneously [52,53,54]. Amphipathic peptides interact with lipids and often tend to adopt secondary structure in the presence of membranes. A high local concentration of CPPs at the membrane surface can lead to the formation of transient secondary structures (lipid-hydrophobic domain of CPP interaction). As most cancer cell surfaces have an overall negative charge, the cellular uptake efficiency of positively charged molecules outperforms that of negatively charged ones [55].

Recently, it has been shown that receptor-mediated internalization is important in the uptake of R_8_. Futaki and coworkers identified syndecan-4 as the proteoglycan that acts as the cell surface target responsible for the uptake of R_8_ via clathrin-mediated endocytosis [34]. In their approach based on photo crosslinking, 17 membrane-associated proteins were found to interact with R_8_ and potentially mediate its internalization. By using knockdown experiments in combination with pharmacological inhibitors, those authors found that syndecan-4 was the membrane-associated receptor preferentially associated with R_8_ uptake. The same group showed how a chemokine receptor (CXCR4) was involved in the macropinocytic internalization of the oligoarginine R_12_ peptide [56]. In summary, the interplay of various mechanisms is involved in the internalization of CPPs, and therefore a number of structural parameters drive this translocation process.

### 3.3. Endosomal Escape

**Figure 2:**
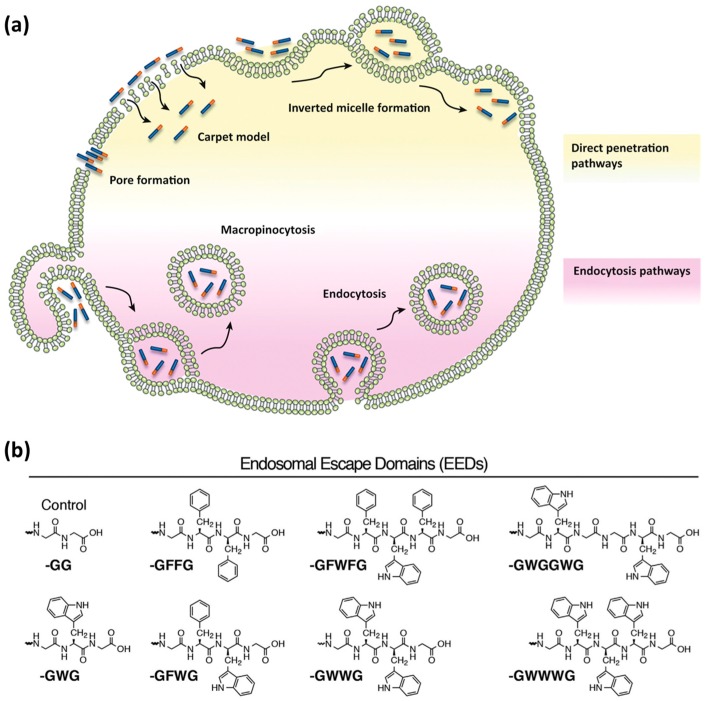
(**a**) Schematic representation of several internalization pathways of cell-penetrating peptides, with emphasis on endocytosis (reproduced with permission from reference [8], Copyright © 2017 Elsevier); (**b**) Peptide sequences used for optimization of endosomal escape domains (and inspired by viruses that insert hydrophobic domains into membranes) taken from reference [59].

Endocytosis is one of the main routes for CPP internalization. It is an energy-dependent process that involves several pathways, classified as the following: (a) micropinocytosis; (b) clathrin- or caveolin-mediated endocytosis; and (c) clathrin- or caveolin-independent endocytosis [8,57]. Endocytosis consists of the uptake of extracellular material through encapsulation into vesicular compartments, called endosomes (Figure 2a). During endocytic internalization, endosomal release (escape) is the rate-limiting step for the efficiency of CPPs to reach intracellular targets [8,58]. A number of strategies to improve endosomal escape have been reported [51,54,59,60,61]. The main examples include the use of acid–labile bonds, endosome-disrupting peptides or polymers, and rupture of endosomal membranes with lasers [51,61].

Inspired by the ability of viruses to destabilize the endosomal lipid bilayers by insertion of motifs containing hydrophobic amino acid side chains, researchers addressed the covalent attachment of hydrophobic so-called ‘endosomal escape domains’ [59,62]. Dowdy and co-workers reported on the design of these domains by exploring combinations of hydrophobic amino acids based on tryptophan and phenylalanine (Figure 2b) [59]. Futaki and co-workers described penetration-accelerating sequences (Pas) [63,64]. Enhanced translocation was obtained by attaching the hydrophobic FFLIPKG sequence to the cationic CPP R_8_. Additionally, stearylation was reported to promote endosomal escape when compared to the unmodified peptide [65]. 

pH-sensitive materials are often designed to have modular behavior depending on the environment [66,67]. CPPs can switch their properties and become membrane lytic when in contact with the lower pH values found in endosomes [55]. An example is the GALA peptide (sequence: WEAALAEALAEALAEHLAEALAEALEALAA), which contains glutamic acid residues [67]. This peptide has an overall negative charge in the extracellular region, which prevents lytic activity. Once in the endosomes and exposed to a slightly acidic pH, these glutamic acid residues are protonated, the charge is reduced, and a helical structure able to lyse the endosomal membranes is formed [55,68].

Recently, the use of endosmolytic peptides, inspired by the spider venom M-lycotoxin, was proposed for intracellular delivery of antibodies [69]. Analogs of the spider venom peptide were designed by introducing one or two glutamic acid residues into the hydrophobic part of the amphipathic helix. In this regard, the membrane lytic activity of the natural cationic venoms decreased, while selective perturbation of endosomes was achieved [69].

Cysteine oxidation state seems to be important for improving efficiency through endosomal escape [25,70]. Cysteines are able to form disulfide bridges in oxidative conditions, resulting in cyclization when they are intramolecular, or giving rise to oligomers or aggregates when they are intermolecular. A study of selective controlled oligomerization that leads to dimer formation and results in enhanced uptake, followed by the reduction to monomers in the cytosol, was reported. However, this behavior was found to be dependent on the cell type used [25].

### 3.4. Influence of Cargo

An important application of CPPs is their use in drug delivery and as diagnostic tools, both in vitro and in vivo [38,71,72]. Recently, CPP-derived therapeutics have reached preclinical evaluations and some have even entered clinical trials [8]. CPPs are able to aid the delivery of therapeutic molecules and/or imaging agents to cells and tissues. The cargo can be introduced through covalent modifications or non-covalently by exploiting weak interactions between CPPs and cargo molecules [73]. Covalent conjugation of CPPs to peptidic drugs, proteins (e.g., antibodies, insulin, etc.), small chemotherapeutic drugs (e.g., doxorubicin, methotrexate, paclitaxel, etc.) and nucleic acids has been reported [38,71]. Non-covalent cargo loading is achieved mainly through the formation of complexes with oligonucleotides [38,71,74,75,76]. The type of cargo, its influence on the internalization mechanisms and/or efficiency of CPPs have been extensively reviewed elsewhere [38,71,72,77].

## 4. Design Strategies

In the language of protein folding, primary structure refers to the amino acid sequence that encodes all the information necessary for folding and assembly into higher organizational levels (secondary, tertiary and quaternary structures). Based on the same principles, short peptides are able to adopt secondary structure segments such as α-helices and β-sheets. These conformational propensities are driven by weak non-covalent interactions such as electrostatic interactions, hydrogen bonding, hydrophobic effect or Van der Waals forces. In addition, short peptide amphiphiles are prone to self-assembly due to intermolecular weak interactions between peptide monomers [37,78]. Today, chemistry offers numerous design opportunities that can be introduced in a programmed and controlled manner. Here, we will describe the CPP design strategies in four sub-sections with increasing structural complexity, based on the classic language of protein folding.

### 4.1. The Importance of the Primary Sequence

The following generic parameters are necessary for CPPs to cross membranes: guanidinium content, hydrophobicity and amphipathicity [79]. The primary structure–function relationship has been extensively studied for arginine-rich CPPs, as described above [19]. Membrane models, as well as membrane extracts, followed by in vivo studies, have often been used to determine sequence and single residue (e.g., positive charge) contributions. However, it is difficult to predict whether a certain peptide will have the potential to cross the membrane on the basis of peptide sequence. Several chemical and physico-chemical properties such as charge, chirality, aromatic and hydrophobic content and often their interplay are important drivers of CPP internalization [15,80]. 

Although effort has been made to understand the contributions of single parameters to CPP passage through membranes, the combinations of charge, guanidinium, hydrophobicity and aromaticity are often explored to obtain more efficient CPPs [81]. Recently, synthetic mimics of CPPs (CPPMs) were developed to control the contribution of charge, hydrophobic functionality, π-electronics and helicity [81]. Ring-opening metathesis polymerization was used to synthesize these mimetics, yielding polypeptides carrying various functionalities of interest. These synthetic polymers are valuable tools for studying CPP–membrane interactions and for elucidating the contribution of individual parameters of the translocation process. The advantage of such polymers is that the desired variability can be introduced in the primary structure in a programmed manner.

**Figure 3:**
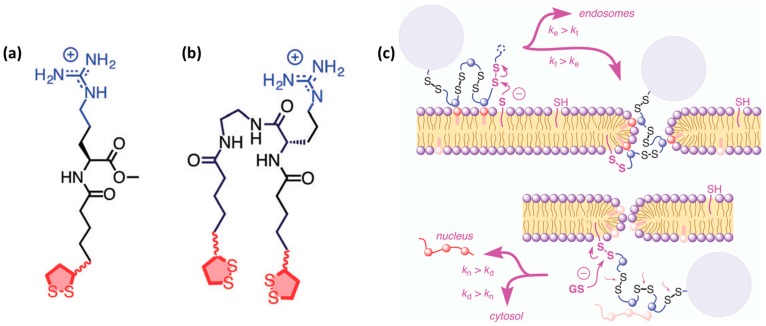
(**a**,**b**) structures of the most promising examples of cell-penetrating polydisulfides containing guanidinium groups and a polydisulfide backbone, taken in part, with permission from reference [13] (Copyright © 2015, Royal Society of Chemistry). (**c**) Schematic representation of the polydisulfide dynamic covalent chemistry on the cell surface and their cell entry by thiol-mediated uptake, followed by reductive depolymerization by glutathione (*k_e_* = rate of endocytosis, *k_t_* = rate of translocation, *k_d_* = rate of depolymerization, *k_n_* = rate of nuclear uptake), taken from reference [94].

***Cationic and arginine-rich CPPs*.** Peptide sequences rich in cationic amino acid side chains hold great promise in the CPP field [19,20,64,82,83]. Following the discovery of Tat, which contains nine arginine and two lysine residues, it is not surprising that the simplest CPP mimics were oligoarginines. In the early 2000s, the correlation between high levels of cell uptake and inclusion of cationic residues in the CPP design was confirmed [84]. Since then, interest has focused on studying polycationic compounds with a variety of scaffolds containing repeating arginine residues, which promote cell penetration. Positive charge can also be introduced through other amino acid side chains, such as lysine and histidine. However, polyarginines outperformed polylysines and polyhistidines [17,85]. Therefore, polyarginines are also the most studied CPPs in terms of mechanism of internalization and introduction of designs. Subsequently, it has been noted that the efficiency with which linear polyarginines are taken up differs depending on sequence length and also on the number and position of arginine residues in the sequence [19,34]. The guanidinium group has the capacity to form bidentate hydrogen bonds with membranes and it is therefore relevant for membrane translocation (Figure 3a) [86,87].

**Figure 4:**
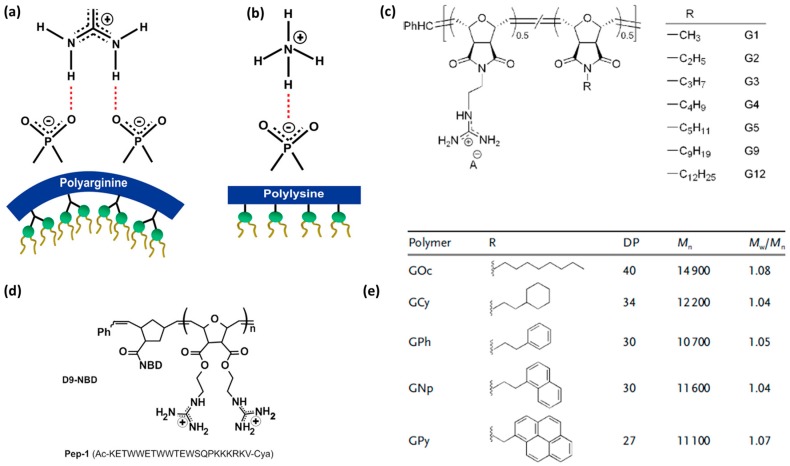
Schematic representation of the (**a**) bidentate and (**b**) monodentate hydrogen bonding interaction of the guanidinium group of the arginine and of the lysine side chain, respectively, with the membranes, taken in part from reference [87]; (**c**) Example of CPP mimetics containing a combination of guanidinium and aliphatic groups of various lengths (from methyl to dodecyl), used to improve internalization efficiency by mimicking the hydrophobic counterion effect (reproduced with permission from reference [98], Copyright © 2011 Wiley) (**d**) Example of CPP mimetic based on the incorporation two guanidines per repeat unit synthesized for a comparison study with a lysine-rich CPP, Pep-1, taken in part, with permission from reference [88] (Copyright © 2015 Elsevier); (**e**) Examples of aromatic functionality introduced in guanindine-containing CPP mimetics, including octyl, cyclohexyl, phenyl, naphthyl and pyrenyl (DP = degree of polymerization, *M*_n_ = apparent molecular weight, *M*_w_/*M*_n_ = polydispersity index), reproduced with permission from reference [97] (Copyright © 2012 Wiley).

In addition, non-natural amino acids (d-stereoisomers) and/or synthetic mimics containing guanidinium moieties or other positively charged chemical entities have been explored [21]. Synthetic mimics are advantageous as various chemical entities can be introduced in a controlled manner. Other advantages include their resistance to proteases and the possibility to study specific contributions of single sequence components. An example is a comparative study in which the authors evaluated a lysine-rich CPP, namely Pep-1, against the guanidinium-bearing CPP mimic [88]. For this purpose, a guanidinium-rich 9-mer was synthesized by ring opening metathesis polymerization, with two guanidines per repeat unit, designed to mimic Tat (Figure 4d). The results revealed that the mimic showed better performance than Pep-1 in terms of transport efficiency when using vesicles as membrane models [88]. A possible explanation for increased efficiency is that guanidinium groups show higher affinity than lysine for hydrophobic anions. Interestingly, this system enabled the study of defined, independent variables introduced on demand [88].

Peptoids are reported to be protease-resistant alternatives to well-known CPPs [89,90]. Peptoids are peptidomimetic molecules in which the side chain is introduced on the nitrogen atom of the peptide chain rather than on the α-carbon. Peptoids do not tend to assume secondary structures as the backbone amine groups are substituted and therefore not able to participate in hydrogen bonding. Nevertheless, examples of cell-penetrating peptoids (CPPos) have been reported [90]. Depending on the interplay of charge and lipophilicity, the cellular uptake/endosomal release efficiency of peptoids and specific organelle (e.g., mitochondrial) localization has been achieved when a lipophilicity threshold was reached. Some authors argue that the introduction of novel cationic side chains other than lysine and guanidine in the structure of peptoids would increase their performance [89]. For this purpose, peptoids containing polyamines, aza-crown ethers, or triphenylphosphonium ions were designed [89]. These novel CPPos showed stability against enzymatic degradation, in addition to cell-penetration properties.

Interestingly, Kelley and co-workers described an example of CPP design to obtain peptides with mitochondrial localization [91]. Two main parameters were included in the design of MPPs (mitochondria-penetrating peptides), which have a positive charge and lipophilic character. Positive charge was introduced through lysine and arginine while phenylalanine and cyclohexylalanine were used to provide lipophilicity [91]. The increased lipophilicity of the triple charged CPPs was the driving force for mitochondrial rather than nuclear localization. A lipophilicity threshold (log P values higher than −1.7), as well as a charge-lipophilicity balance, was essential for mitochondrial localization. When compared to Tat, the same penetration efficiency was observed but with differential intracellular localization, being nuclear or mitochondrial. Recently, a review on MPPs was published by the same group [92].

***Cell-penetrating polydisulfides.*** A conceptually innovative way for improving cell penetration is the use of cell-penetrating polydisulfides (CPDs) [93]. Polydisulfides, obtained by ring-opening disulfide-exchange polymerization, are guanidinium-rich compounds in which the peptide backbone is replaced by a polydisulfide one [94]. The topic has been reviewed in detail elsewhere [13]. Briefly, CPDs are interesting as they are involved in dynamic covalent chemistry on the cell surface and enter cells by thiol-mediated uptake (Figure 3c) [94]. The most promising CPDs are those shown in Figure 3a,b. They are advantageous as they undergo reductive depolymerization by glutathione upon cell internalization (Figure 3c) and do not get trapped in endosomes. They are able to enter HeLa and MCF-7 cells and efficiently deliver proteins [95]. Their uptake was determined by flow cytometry at a concentration of 500 nM. In addition, it has been observed that they show identical intracellular localization, regardless of whether they carry a cargo, thereby suggesting that their cell penetration is not perturbed by the presence of large biomolecules [95]. An increase in CPD length affects depolymerization kinetics [96], which in turn also influences their preferential intracellular localization in endosomes, cytoplasm and nucleoli. This system was further developed to obtain double-labeled CPDs with a FRET pair at opposite ends of the polymer. These CPDs were shown to enter cells, although they were split into two fragments at the outer leaflet of the membrane during uptake [94]. For a comprehensive overview of this topic, please refer to the review by Matile and co-workers [13].

***The role of hydrophobicity and aromaticity.*** In addition to cationic moieties, lipophilic ones are also important for cellular uptake [97,98,99]. One proposed mechanism implies that hydrophobic counterions form around the guanidinium-rich backbone of CPPs [98]. The counterion effect was first shown as the hydrogen bonding interaction between arginine residues and membrane components, to form complexes that are able to cross membranes [86,100]. This strong bonding of counterions was not observed for lysine-rich peptides, thereby highlighting the relevance of the guanidinium group (Figure 4a,b) [87,100]. The formation of the counterion complexes by the hydrophobic parts of the CPP sequence is referred to as the self-activating property of these peptides [98]. Therefore, studies exploring hydrophobicity, but also individual aliphatic (Figure 4c) and aromatic (Figure 4e) functionality contributions, were needed. Some studies on CPP mimics confirmed that aromatic activators outperform aliphatic ones [97,101].

Hydrophobicity can be achieved by adding aliphatic or aromatic moieties. On the one hand, lipidation is obtained by attaching hydrocarbon chains of different lengths to the N-terminal of known CPPs [39]. Alkylation is a conventional way to increase internalization through enhanced hydrophobic interactions with the membrane [39,65,82]. Hydrophobicity can also be introduced by incorporating amino acids with alkyl side chains into the peptide sequence. By introducing alky moieties of different lengths into CPP mimetics, Tew and co-workers reported a three-fold improvement in activity when moving from methyl to butyl side chains [98]. Figure 4c shows a list of the alkyl chains used for that study. Interestingly, when silaproline (γ-dimethylsila-proline) was used instead of proline to obtain a new polyproline PPII helical CPP, better internalization efficiency was observed. According to the authors, this observation is attributable to the overall increase in hydrophobicity [79]. 

Alternatively, hydrophobicity can be achieved by adding aromatic amino acid residues, such as tryptophan, phenylalanine and tyrosine [97]. These side chains also account for the contribution of aromaticity. According to Wimley and White, aromatic residues have favorable free energies of insertion at the bilayer interface [99]. To study the introduction of both aliphatic and aromatic amino acids in CPP design, CPP mimics were used to report on individual contributions, i.e., hydrophobicity or aromaticity. As previously reported, the mimics were obtained by ring-opening polymerization. Pyrene, coronene and fullerene were shown to trigger guanidinium ion activation, thereby allowing the passage of CPPs across membranes and an increase in the uptake of polyarginines [101]. Later, the specific role of aromatic functionality compared to overall hydrophobicity was confirmed by Tew and co-workers [97]. The authors designed a new series of CPP mimics to determine whether the aromatic functionality provides better transduction efficiency than the aliphatic one, while maintaining the same relative hydrophobicity [97]. 

Aromatic functionality was found to contribute to CPP internalization. Because aromatic groups have the ability to π–π stack with membrane proteins containing aromatic residues, they may contribute to promoting and/or stabilizing the interactions of CPPs with membranes and help translocation. In this regard, π–stacking was studied on vesicle-based models [81]. This concept was extended to the analysis of the influence of various π-interactions (π–π, π–cation, π–anion, and π–polar) on the internalization. For this purpose, monomers containing aromatic rings substituted with electron-donating and -withdrawing groups were used to show the effect of changes in quadrupole moments on these systems [81]. 

The examples mentioned reflect the versatility of design possibilities of the primary structure, i.e., at the sequence level. When introduced in a programmed manner, these design strategies contribute to elucidating the individual structural components necessary for uptake.

***Chirality.*** The susceptibility of conventional CPPs based on natural L-amino acids to proteases is a major drawback for their application in vivo. Various strategies to increase stability have been introduced in the design of the primary structure of CPPs. These are based mainly on the use of non-natural amino acids, such as all d-stereoisomer versions of CPPs and more recently on l- and d-combinations [102,103,104]. Cyclization has also been explored in these terms [105]. Results showed that d-peptides were efficient in increasing stability; however, they had similar or often lower CPP efficiency. Zhang and co-workers showed that the internalization efficiency is affected by the number of d-arginine residues in the peptide sequence [102]. It has also been observed that the uptake of l- versus d-amino acid CPPs occurs in a cell-dependent manner and that it is dependent on two factors, namely heparin binding and initiation of internalization [106]. Brock and co-workers explained that chirality has an effect on the initiation of internalization in selected cell types, thereby providing useful knowledge for potential future stereochemistry-dependent targeting of cells [106]. Another report showed that multiple substitutions of l- to d-amino acids alter the internalization efficiency of anionic and amphipathic CPPs in a cell-dependent manner and that the position of d-substitution within the peptide is a key parameter [104]. Interestingly, when the d-substitution was responsible for altering the α-helix to β-sheet ratio of anionic CPPs, cell entry was altered [104]. In addition, the uptake efficiency effect of all d-stereoisomers was shown in vivo by Giralt’s group, where polyproline-based CPPs exhibited enhanced protease-resistance and similar internalization behavior compared to all l-versions [103]. 

The contribution of chirality to internalization efficiency remains unclear; however, the main advantage of using d-stereoisomers in CPP design is proteolysis resistance. Similar penetration profiles of all d-stereoisomers might be achieved through longer exposure times. 

### 4.2. Flexible Amphipathic CPPs

Since the early 1950s, when Pauli, Corey and Crick elucidated secondary structures such as β-sheets [107] and β-hairpins, α-helices and coiled coils in proteins [108], great advances have been made in the understanding of protein folding. The distinct natures of amino acid side chains determine protein behavior and folding characteristics.

**Figure 5:**
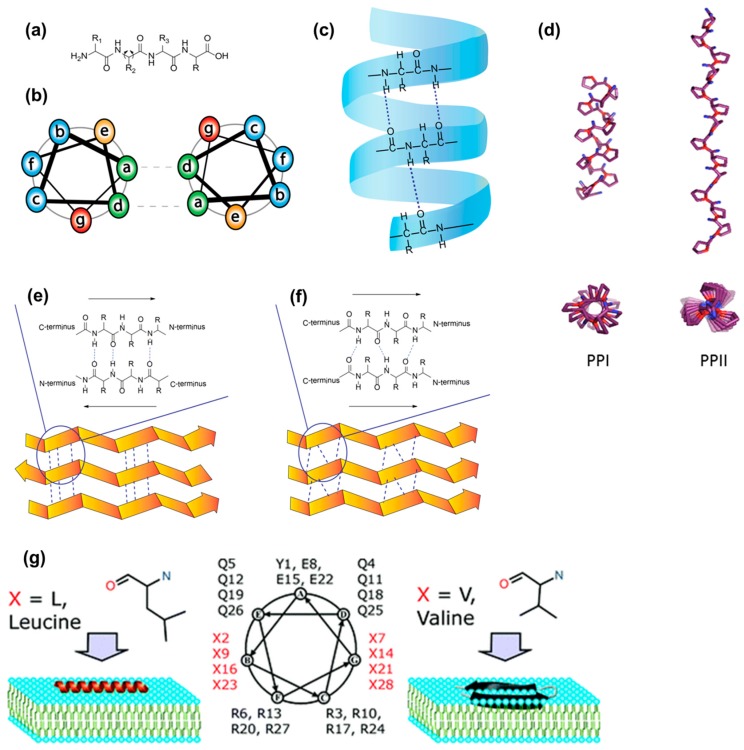
Schematic representation of (**a**) primary peptide sequence with dihedral angles; (**b**) heptad units: (*abcdefg*)_n_ where the *a* and *d* (green) are hydrophobic amino acids, *e* and *g* (orange and red) are oppositely charged amino acid residues, and *b*, *c* and *f* (blue) represent the solvent-exposed amino acids; (**c**) α-helix structure indicating hydrogen bonding; (**d**) Structures of PPI and PPII reprinted with permission from reference [113] (Copyright © 2017, American Chemical Society); schematic representation of (**e**) anti-parallel and (**f**) parallel β-sheets found in proteins and synthetic peptides. (**g**) Schematic representation α-helical or β-sheet folds that form upon the interaction of CPPs with membranes, where even small changes in the primary sequence result in different folding behavior, reproduced with permission from reference [112] (Copyright © 2010, American Chemical Society).

α-Helical secondary structures (Figure 5c) form when the peptide backbone of a single amino acid chain coils as a result of repeating backbone dihedral angles (Figure 5a). In this conformation, each amino acid residue hydrogen bonds with the amino acid residue, which is four positions further along the chain. Assemblies based on α-helical conformation are characterized by an α-helical coiled coil motif with the repeating heptad unit (*abcdefg*)_n_, where each letter indicates a precise position and accommodates a specific amino acid type (Figure 5b) [109,110]. In *a* and *d*, which stand for positions one and four respectively, only hydrophobic amino acids can be accommodated, thus creating the required spacing between hydrophobic units. The fifth and seventh position (*e* and *g*), closely located on neighboring helices, usually contain oppositely charged amino acid residues. This electrostatic attraction can contribute to the stabilization of the helices. The other three positions (*b*, *c* and *f*) are the solvent-exposed residues and they have fewer restrictions, thus several residues can be explored for them. α-Helical antimicrobial cationic peptides that tend to assume a bioactive helical conformation when in contact with cell surfaces are an example [111]. Administered as soluble inactive forms, they fold when in contact with negatively charged membranes. In addition, well known CPPs, such as Pep-1, MPG, TP10 and SAP, were shown to adopt inherent helical structures or assume helical conformation when in contact with cell membranes (Figure 5g) [112]. Their folded states promote membrane permeation [80].

Polyproline helices PPI and PPII are a type of protein secondary structure composed of repeating proline residues (Figure 5d) [113]. They consist of all *cis* or all *trans* conformations of the peptide bond, respectively. The PPI helices form preferentially in organic solvents and are right- handed. On the other hand, when exposed to aqueous environments, polyprolines tend to form left-handed PPII helices. Interestingly, PPII helices have cell-penetration properties [114]. 

In proteins, β-sheet-like secondary structures are dominated by inter-chain hydrogen bonding between closely aligned carbonyl and secondary amino groups on the peptide backbone. They can be formed by proteins, peptides of de novo design, β-hairpins, and aromatic and aliphatic peptide amphiphiles. The formation of β-sheets can occur in two ways. Parallel β-sheets form when the direction of each N (N-terminus) to C (C-terminus) peptide backbone is in the same direction (Figure 5f). Anti-parallel β-sheets are observed when the peptide backbones are parallel but the direction of the peptide chains alternates, going from N to C and from C to N (Figure 5e).

In short peptide sequences, β-sheet formation can be obtained by alternating hydrophobic with hydrophilic residues, by introducing aromatic moieties, or by basing the design on known β-sheet-forming moieties. β-sheet-forming peptides based on EAK and RADA peptide sequences, where hydrophobic amino acids residues are alternated with hydrophilic charged ones, have been studied primarily for regenerative medicine applications [115,116,117]. Peptides that spontaneously form β-structures when in contact with cell membranes have been proposed for anticancer applications. Based on knowledge of antimicrobial peptides, Schneider and co-workers presented a cationic peptide (SVS-1) that disrupts cancer cell membranes by exploiting their aberrant phospholipid distribution [118]. This peptide was observed to fold on the surface of cancer cells while it remained unfolded in aqueous solution. It is capable of disrupting the membrane only by adopting the amphiphilic, β-hairpin structure. This membrane-induced folding, driven by the electrostatic interaction between the positively charged peptide and the negatively charged membrane surface of cancer cells, is essential for its anticancer activity [118].

***Folding and secondary structure of CPPs to control translocation.*** Two main parameters to be considered when studying the effect of secondary structures on CPP translocation efficiency are (a) affinity of CPPs for membranes and (b) their folding capacity in the presence of membranes. These two parameters are interconnected as they are based on the same principle of weak interactions. Their contribution is essential to understand the driving forces of CPP internalization and to improve the design of future CPPs. As already mentioned, the CPP-phospholipid interaction in membranes is based on the interplay of weak interactions, namely electrostatic interactions, hydrophobic effect and hydrogen bonding. As a result of these interactions, CPPs often fold and assume secondary structures. Their ability to fold is also closely related to their amphipathic nature. The folding properties (inherent, i.e., primary sequence-related or membrane-induced) of CPPs have been studied in detail in membrane models to better understand the role of the *interfacial properties* and *conformational state of these peptides* on internalization efficiency. Adsorption experiments at the air–water interface imply that α-helical- and β-sheet-like peptides differ in amphipathic character [15]. Helical CPPs were found to be more amphipathic than β-sheet ones. In addition, it has been noted that internalization may differ in function of amphipathicity [15].

Swiecicki and co-workers rationalized the affinity of CPPs for the membranes based on peptide *amphipathicity* [9]. They reported on translocation mechanisms of well-known CPPs, such as penetratin, Tat, R_9_, R_6_/W_3_ (RRWWRWRR), Pep-1 and TP10 (AGYLLGKINLKALAALAKKIL) using vesicles as model membranes [9]. All examples described were based on cationic peptides with charges from +4 to +9 that show variable or no amphipathicity. The following CPP classification into three subgroups was proposed: *primary amphipathic*, *secondary amphipathic* and *non-amphipathic*. Primary amphipathic CPPs (pep-1 and TP10) are characterized by a primary peptide sequence where hydrophobic and cationic residues are segregated at the primary structure level. On the other hand, secondary amphipathic molecules, such as penetratin and R_6_/W_3_, are made of hydrophobic and cationic residues that have an amphipathic character only when the CPP is in the folded state. When in α-helical conformation, secondary amphipathic molecules are able to expose the hydrophobic and the cationic side, while this repartition of the residues is not achieved at the primary sequence level. Finally, the unstructured group comprises polycationic CPPs such as R_9_.

An example of a tryptophan-rich secondary amphipathic CPP is CADY (sequence: Ac-GLWRALWRLLRSLWRLLWRA-cysteamide). This 20-mer was designed by combining aromatic tryptophan and cationic arginine residues into a secondary amphipathic CPPn [119]. When in contact with cell membranes, it adopts a helical conformation by exposing hydrophobic residues on one side and the cationic ones on the other side of the helix. 

Divita and co-workers studied the *structural state* and conformational plasticity of 10 well-known CPPs in various environments (membrane models) [15]. They observed α and β structures in the presence of phospholipids. The ability of CPPs to interact with membranes via electrostatic interactions often triggers conformational transitions from random coil disordered states to more organized α-helical or β-sheet conformations [35]. Therefore, on the basis of their capacity to form secondary structures in the presence of phospholipids, CPPs were divided into the following subgroups: (a) *random coils*, (b) *β-structures* and (c) *α-helical conformations* [9,15]. The main structural transitions that occur when CPPs are exposed to new environments (presence of phospholipids) have been summarized elsewhere [9,15]. Briefly, Tat and R_9_ are unstructured in buffered solutions, while other CPPs (Penetratin, R6/W3, Pep-1 and TP10) show mixtures of conformations or low levels of secondary structure. At high concentrations, only Pep-1 presents a helical conformation in water.

Since Tat and R_9_ are of hydrophilic polycationic nature, they remain unstructured regardless of the environment, and they translocate as random coils. R_6_/W_3_, Pep-1 and TP10 are in the helix group as they are random coils in the presence of zwitterionic phospholipids but can fold to form helices in the presence of negatively charged phospholipids. These peptides translocate when in helical conformations. Penetratin is random coiled in the presence of zwitterionic phospholipids. However, its behavior is determined by experimental conditions, i.e., phospholipid charge density and CPP/phospholipid ratio. Penetratin is part of the β-sheet group as it undergoes a random coil to β-sheet transition at high CPP/phospholipid ratios. In addition, it was found to fold into helices in the presence of low amounts (20%) of anionic head groups and to transition from α-helical conformation to β-sheets when the ratio of CPP/phospholipid or negative phospholipid content increases [120].

The structural polymorphism of penetratin and transportan was examined using Boltzman–Stochastic algorithms to assess the influence of the environment on their behavior in proximity to membranes [121]. For this purpose, models were developed for hydrophobic and hydrophilic environments and compared with experimental NMR data. In the case of penetratin, the tryptophan residues were replaced by phenylalanine to obtain mutants with increased helicity in a hydrophilic environment. For transportan, deletion analogs were modeled. In both cases, the structural polymorphism resulted essential for maintaining cell permeability [121].

A specific class of CPPs is based on polyproline secondary structures. Proline presents unique structural properties as it is the only one of the 20 genetically encoded amino acids to have a secondary amine chain. For this reason, it does not participate in intra- or inter-molecular hydrogen bonding. CPPs forming polyproline-type secondary structures were discovered by Giralt and co-workers [24,122,123]. These CPPs show a common (VXLPPP)_n_ general structure [123]. A library of polyprolines was synthesized to study the effect of primary and secondary amphipathicity on penetration efficiency ((VXLPPP)_n_ with X = H, K, R; n = 1,2,3) [79]. It was concluded that internalization of such compounds is due to the amphipathicity of the secondary structure rather than of the primary sequence. The best performing CPP was (VRLPPP)_3_ and it was named sweet arrow peptide [24]. To increase its efficiency, modifications were introduced by adding fatty acid moieties to the N-terminus of the sequence [39] or by mutating one of the prolines to a silaproline [79]. In addition, an all d-version of the sweet arrow peptide was prepared to obtain CPPs with greater resistance to proteases [103].

A chemically modified version of the sweet arrow peptide (VRLPPP)_3_, in which arginine was replaced by glutamate residues, presents PPII helical secondary structure and carries a net negative charge. Surprisingly, this first-in-class anionic CPP exhibits the same cell-penetration activity as SAP, its cationic counterpart [124]. In addition, when the Ac-CGGW sequence was added to the N-terminus of SAP(E) as a binding motif, immobilization to surfaces or attachment to biologically active substances was achieved [125]. This modification did not influence the uptake rates of the peptide, and the internalization was due to polypeptide aggregation on the cell surface, followed by endocytic uptake [125]. The studies were based on model membranes in both lipid mono- and bi-layer configurations. It was found that the initial binding is induced by structural changes of the peptide (in bulk, it assumes a PPII helical structure that undergoes a structural transition at membrane interfaces either by refolding or self-aggregating) [125].

Interestingly, a β-strand-forming CPP based on the γ-polyproline backbone was reported [126]. After designing a non-natural proline-derived γ-peptide backbone using a cyclic conformationally constrained *cis*-γ-amino-l-proline as monomer, γ-peptides with the ability to adopt secondary structure in solution were obtained. The secondary structures identified by both NMR and CD analysis suggested the formation of an isolated H-bond ribbon, resulting in a short β-strand [127]. Alkylated, acylated and guanidinylated variants were examined for their capacity to enter cells. CPPs with the ability to enter cells via endocytic pathways were obtained by functionalizing the α-amine with amino acid side chains such as alanine, leucine and phenylalanine. These new unnatural short oligomers showed improved solubility, protease resistance, and lower toxicity when compared to Tat [126].

**Figure 6:**
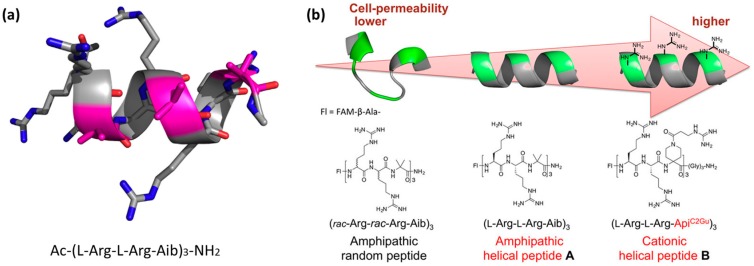
(**a**) Calculated minimum energy conformation of a short Aib-containing CPP, where helix stabilization is induced at primary structure level, reproduced with permission from reference [129] (Copyright © 2014 Elsevier); (**b**) Schematic representation of the relationship of secondary structure properties of CPPs and their cell permeability in various environments, taken from reference [130].

***Helicity induction or stabilization through sequence design.*** Unlike the examples based on unstructured CPPs that adopt a bioactive conformation when in contact with cell surfaces, helicity can be obtained through the design of primary sequences. Helicity can be programmed into the primary sequence by using a natural non-coded α-aminoisobutyric acid (Aib) [128]. CPPs that contain Aib, often used to stabilize peptide helical structures, at the primary sequence level were reported [129]. To gain knowledge on the control of the secondary structures of these short peptides, the authors developed a number of amphipathic nonapeptides containing l-Arg, d-Arg, and the achiral Aib residues (Figure 6a). They observed that the helical structure contributed to the ability of the peptide to cross membranes. The peptides that formed stable α-helical structures were more efficiently internalized into Hela cells than the non-helical ones [129].

To assess whether the CPPs are able to change their secondary structure in response to the environment, this study was extended to the introduction of L-proline or the modified guanidinyl l-proline (Figure 6b). In this regard, modular CPPs were designed based on the ability of proline residues to sense environments (hydrophilic and amphipathic) [130]. The authors reasoned that the conformational change from random coil to helical structure when in contact with membranes contributes to enhanced endosomal escape. The peptide with the best performance, namely the one containing guanidinium-modified l-proline, was further explored for plasmid DNA transport into HeLa cells [130].

**Figure 7:**
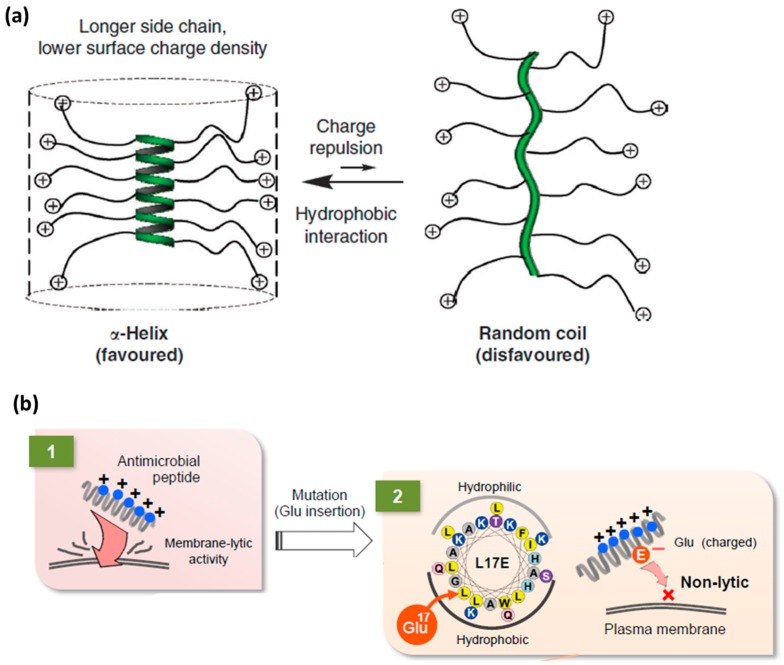
Schematic representations of (**a**) introduction of helicity at the primary sequence level, through addition of hydrophobic, alkyl chains of different lengths to fine tune the folding of random coiled, positively charged CPPs, reproduced from reference [48] (Copyright © 2011, Nature Publishing Group) and; (**b**) modulation of lytic properties of peptides through replacement of specific residues in the hydrophobic part of the amphipathic helix, reproduced from reference [69] (Copyright © 2017, Nature Publishing Group).

As previously mentioned, guanidinium-rich peptides lack the ability to fold and adopt helical structures. Therefore, polyarginines adopt a random coil configuration at physiological pH. Interestingly, when both helicity and hydrophobicity are included in their design, helical arginine-rich mimics are obtained, showing membrane activity two orders of magnitude higher than that of Tat [80]. Polyarginine analogs with a polypeptide backbone obtained via ring-opening polymerization and designed to contain guanidinium side chains placed at fixed distances from the peptide backbone were shown to adopt a stable α-helical conformation [80]. The addition of alkyl chains of different lengths (using click chemistry) led to alterations of hydrophobicity. Higher alkyl chain content, from –C_3_H_7_ to –C_6_H_13_, resulted in enhanced helical propensity, which contributed to enhanced membrane permeability (Figure 7a) [48]. 

The importance of the concomitant effect of helicity, polypeptide backbone length and side chain hydrophobicity on membrane permeability was reasoned. The helical polyarginine mimics were labeled with rhodamine and their membrane permeability in carcinoma (HeLa) cells, fibroblasts (3T3-L1) and macrophages (Raw 264.7) was examined. These helical mimics outperformed the cell penetration efficiency of Tat and R_9_ by 1–2 orders of magnitude, as determined by spectrofluorimetry, and were able to deliver DNA and siRNA to mammalian cells. This example shows how helicity stabilization (secondary structure) through primary sequence modification is a useful approach to obtain CPPs with higher activity [80]. 

Based on the principle of polyproline folding, small proteins with intrinsic cell-permeability can be designed. These small proteins (36-residue polypeptides) with minimal cationic content within a PPII-type helix can cross membranes [47,131]. They can be stabilized by reducing the cationic content. Therefore, a minimal content of arginine residues has to be found that guarantees cell permeability while preserving protein stability. In this design strategy, the arginine residues were located on the solvent-exposed side of PPII helices.

Similarly, Futaki and co-workers designed a helical CPP by developing analogs of a cationic cytolytic peptide that presents helical secondary structure, already described in the endosomal escape section [69]. The goal was to maintain helicity while reducing the positive charge by replacing small hydrophobic residues such as leucine, glycine or alanine in the hydrophobic part of the amphipathic helix with glutamic acid ones (Figure 7b) [69]. 

### 4.3. Rigidity as an Emerging Concept in CPP Design

There is a concentration threshold, influenced by sequence length, that promotes one internalization route over another [21,38]. Similarly, the structure of the scaffold is important to achieve optimal spatial distribution of guanidinium groups for multivalent interaction with membranes. Cyclization is a means of maximizing membrane contacts as it leads to a more controlled distribution of positive charge when compared to free arginines present on linear CPPs [132]. Cyclization has been shown to increase the kinetics of Tat internalization (cyclic Tat vs. linear Tat) [21]. In addition, multivalency has been introduced using dimerization and transient (reversible) cyclization through di-sulfide bonds or triazole bridges [49,133,134,135].

**Figure 8:**
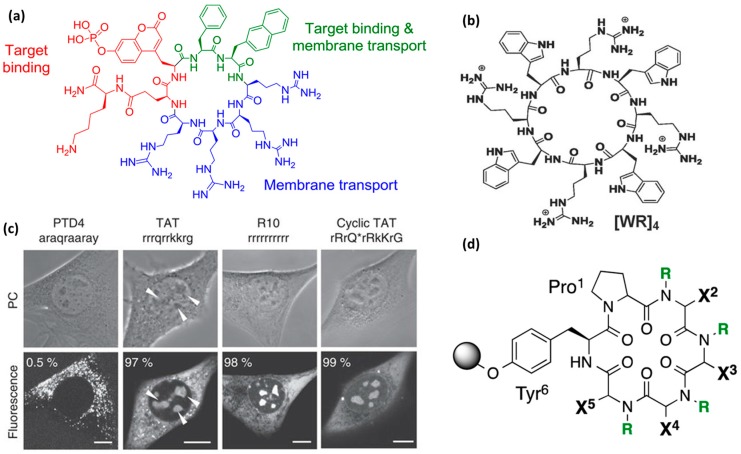
(**a**) Example of the design of cyclic CPPs containing different domains, such as repeating arginine units, a hydrophobic region to facilitate membrane interactions, and a target binding region (reprinted with permission from reference [140], Copyright © 2013, American Chemical Society); (**b**) Chemical structure of a different example of cyclic CPP design, based on alternating arginine with tryptophan residues, with the [WR]_4_ candidate showing the best performance, used with permission from reference [136] (Copyright © 2011 Wiley); (**c**) Phase contrast and confocal images of live-cell transduction ability of fluorescently labeled CPPs: PTD4, Tat, R_10_ and cyclic Tat were internalized into living mouse myoblast cells with different efficiency, cyclic Tat being the most efficient (taken from reference [21], Copyright © 2011, Nature Publishing Group); (**d**) Generic structure of the library of geometrically diverse cyclic hexapeptides (based on l- and d-stereoisomers), reprinted with permission from reference [137], Copyright © 2015, American Chemical Society.

***Head-to-tail cyclization.*** It has been proposed that *cyclization* can be used to confer greater proteolytic resistance and increased penetration efficiency [21,49,60,105,133,136,137,138]. Parang and co-workers reported a study on 11 amphipathic cyclic peptides composed of hydrophobic (W, F, L) and charged residues (K, R, E) to obtain an optimal amphipathic CPP able to undergo intramolecular and intermolecular interactions [136]. All the combinations evaluated were homochiral L-cyclic peptides. The best internalization results were obtained with [WR]_4_ (Figure 8b) and [WR]_5_. These peptides were further analyzed as delivery tools for a peptide template and a model drug (doxorubicin). The peptides did not show cytotoxicity up to 50 µM (MTT) in a leukemia cell line (CCRF-CEM), colon adenocarcinoma (HT-29), breast carcinoma (MDA-MB-468) or human ovarian adenocarcinoma (SK-OV-3).

An example of the use of cyclization to introduce greater peptide structural rigidity was reported by Cardoso and co-workers [21]. In that study, the efficiency of a retro-enantio version of linear Tat (rrrqrrkkrg) and R_10_ to enter living mouse myoblast cells was compared with that of cyclic Tat (K-rRrQrRkKrG-E) (Figure 8c). In the structure of cyclic Tat, K- and E- amino acids were added to the linear Tat sequence to allow cyclization and obtain a ring with the same overall charge as the native form. This resulted in improved transduction kinetics of arginine-rich cyclic peptides, which presented lower degrees of freedom when compared to the linear counterparts that have higher flexibility. In addition, cyclization caused the guanidinium groups to remain in maximally distant positions and assume a controlled spatial distribution within the structure. This resulted in enhanced contacts with the membrane and therefore faster penetration. Previously, introducing spacers such as glycines into polyarginine CPPs and thus increasing the distance between guanidinium groups led to improved cell permeability [139]. Similarly, this approach allows the distances between guanidinium groups to be increased, but without the need to modify peptide sequences responsible for cell penetration [21]. 

Pei and co-workers presented another example of cyclic arginine-rich CPPs internalized by endocytosis [60]. These compounds showed higher proteolytic stability compared to Tat and R_9_. Their cellular uptake increased upon cyclization [60]. Cyclic peptides with a general formula (cFΦR_4_, where Φ stands for l-2-naphthylalanine) were synthesized using varying design parameters, such as sequence lengths, stereochemistry or a combination of the two. Cellular uptake efficiency in HeLa cells was assessed by flow cytometry. All the analogs showed internalization properties. From this study, it was concluded that two aromatic and three arginine residues were enough to obtain functional CPPs (Figure 8a). However, the uptake efficiency was also closely related to the specific peptide sequence. Cyclization, and therefore the introduction of structural rigidity, most probably led to the achievement of a proper spatial arrangement of guanidine groups and hydrophobic side chains, which were critical for internalization efficiency. Furthermore, pharmacokinetic studies were performed in vivo following intravenous injection or oral administration of cFΦR_4_ CPPs in ICR male mice. Oral bioavailability of 4% was observed, thereby confirming their metabolic stability and opening up possibilities for oral delivery [60].

Similarly, Nielsen and co-workers designed changes in a natural product based on the cyclic heptapeptide sanguinamide A in order to increase its oral bioavailability [105]. The optimization was based on NMR studies. l-amino acids, namely phenylalanine, alanine, isoleucine, two prolines and an isoleucine-thiazole dipeptide derivative, were used to obtain a rigid scaffold through the formation of the heterocycle. Rigidity was further reinforced through bulky hydrophobic (tertbutyl glycine) side chains and by favoring intramolecular hydrogen bonds. The introduction of constraints resulted in greater oral bioavailability in rats [105].

*A combinatorial library approach* was used to discover cyclic peptides with increased cell permeability [137]. A library, inspired by natural products, of more than 1000 compounds based on cyclic hexapeptides with a general structure Pro-(Xaa)_4_-Tyr was prepared (Figure 8d). Stereochemistry and N-methylation were the two parameters used to obtain backbone geometries that resulted in different conformational preferences. l- and d-Leu and Me-Leu amino acids were explored in positions X_2_–X_5_. By introducing amino acids with variable side chains in X positions, the effect of lipophilicity and backbone flexibility was assessed. It was found that side chain orientation and steric factors drive the internalization properties of these cyclic CPPs. The advantage of this study is the possibility to explore the relationship between conformation and cell permeability. Although it constitutes an interesting example of cyclic CPPs, their permeability (passive diffusion) was assessed by PAMPA assay only. Activity comparison with standard (classical) CPPs has not been reported for this set of CPPs to date [137].

Interestingly, the triazole bridge was proposed as a means to control the penetration of cyclic peptides into membranes [49]. Three cyclic peptides were obtained by cyclization of a previously developed linear CPP, called sC18 (sequence: GLRKRLRKFRNKIKEK), which is based on CAP18 (sequence: GLRKRLRKFRNK) antimicrobial peptide. In sC18, glycine and lysine residues were replaced by propargylglycine and ε-azidolysine, respectively, to allow cyclization by copper-mediated click chemistry. Triazole bridges were introduced to explore whether secondary structure arrangements could be promoted. sC18 was classified as secondary amphipathic as it assumed α-helical conformation when in contact with artificial lipid membranes [49]. The three cyclic peptides developed differed in the number of arginine residues present within the cyclic structure. In this regard, the influence of spatial distribution of guanidinium groups was explored. Cyclization was obtained by connecting residues at positions 1 + 4, 1 + 8 and 1 + 12 of the linear peptide. The synergistic contribution of backbone rigidification and static arrangement of the guanidinium groups (arginine side chains), together with the conformation, were expected to contribute to enhanced internalization. The peptides were non-cytotoxic in the concentration range of 2–200 µM for breast adenocarcinoma (MCF-7), colon adenocarcinoma (HCT-15), cervical carcinoma (HeLa) and embryonic kidney (HEK-293) cell lines. Their increased uptake depended on the cycle size, with the largest cycle showing the highest membrane activity. Therefore, the 1 + 12 cyclic peptide was used for the delivery of plasmid DNA (in the form of complex), proving to be efficient in transfecting MCF-7 cells.

**Figure 9:**
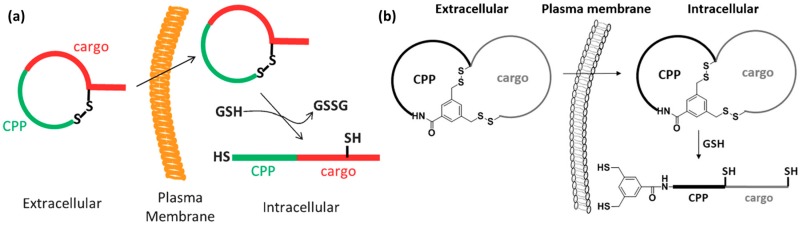
Schematic representations of reversible (**a**) cyclic (cyclization through a disulfide bridge) and (**b**) bicyclic (cyclization through a pair of disulfide bonds) CPPs that undergo reduction upon exposure to glutathione in an intracellular environment, used with permission from references [133] (Copyright © 2015 Wiley) and [138] (Copyright © 2017 Wiley), respectively.

***Disulfide bridges and bicyclization.*** When cFΦR_4_ CPPs were developed, two types of cyclization were proposed. Originally, N-to-C cyclization was reported (Figure 8a) [140]. In addition, cyclization through an intramolecular di-sulfide bond was proposed by modifying the sequence to contain two cysteine residues (Figure 9a) [133]. Both versions were efficiently internalized by HeLa cells and were able to escape endosomes. The advantage of the latter strategy was the reversibility of the cyclization in a reductive environment and increased proteolytic stability. Moreover, Pei and co-workers proposed a bicyclization strategy to increase the uptake of generally impermeable cyclic peptides [134]. The combinatorial library approach was also put forward for bicyclic CPPs, where cFΦR_4_ was fused to over 5 million distinct cyclic peptides to generate a bicyclic cell-permeable peptide library [134]. An important feature of the library design was the use of a rigid and planar scaffold such as the trimesoyl group. This allowed the formation of the desired bicyclic structure, where CPP and cargo moieties were oriented away from each other, thereby minimizing interference. One ring was the previously reported cyclic CPP (cFΦR_4_), while the other contained a ligand for K-Ras, which is a very difficult intracellular target. The general applicability of the cyclization approach was assessed. Two rings were used, one with CPP properties and the other with having the peptide sequence (X_5_) based on 26 amino acids used in different combinations in positions X_1_–X_5_ and containing 10 proteinogenic l-amino acids, six unnatural l-amino acids and 10 d-amino acids [134].

Recently, the same group has reported on a reversible bicyclization strategy achieved through the formation of a pair of disulfide bonds (Figure 9b) [138]. Conformationally constrained bicyclic structures were obtained with the goal to induce the uptake of generally impermeable peptidyl drugs. Another advantage of this approach is increased proteolytic stability. The novelty of the design is the presence of a small CPP ring for enhanced uptake, in addition to a cargo ring able to accommodate peptides of various lengths. For this purpose, two model peptides containing two cysteine residues were designed (RRRRΦF and FΦRRRR). One cysteine was positioned at the C-terminus while the other was placed at the fusion point between the CPP and the cargo. Cyclization was performed on-resin between the two cysteine side chains and the 3,5-bis(mercaptomethyl)benzoic acid was used as a scaffold to obtain a single bicyclic structure. This scaffold was chosen because of its symmetry. Flow cytometry analysis of HeLa cells showed that bicyclic peptides had a greater capacity to enter cells than the CPP cFΦR4, which holds only one cycle. Interestingly, the in vitro treatment of these bicyclic peptides with glutathione led to reduction of disulfide bonds. This observation suggests that these peptides would undergo reduction upon cell penetration [138].

***Multivalency.*** Another parameter to consider is *the multivalency of the system*. One step in this direction is the development of dimeric branched peptides, as shown by Saludes and co-workers [135]. The covalent dimeric branched peptide was designed as an analog of Tat. Controlled dimerization was obtained through bis-Fmoc protected lysine near the C-terminus. Cargo (Cyanine 5) was introduced on the lysine side chain at the C-terminus residue. The uptake in HeLa cells was determined using flow cytometry and compared to that of R_8_, penetratin and linear Tat. The novel branched peptide showed enhanced uptake compared to the controls. Importantly, the non-linear dependence on concentration and uptake at sub-micromolar concentrations was observed for the dimers. In addition, the dimers were non-cytotoxic to Hela cells at a concentration up to 50 µM. A similar trend was observed in primary hippocampal neuronal cells. This example shows how the introduction of multivalency can improve CPP peptides [135]. In addition, the ability to cross membranes may have been promoted as a result of increased local concentration on the membrane surface in a synergistic fashion.

Extended peptides based on oligoarginines were developed through disulfide bridge formation. Based on the knowledge that polyarginines need a minimum number of residues (at least 6) to cross membranes, Lowik and co-workers designed small inactive oligoarginine fragments (R_4_) that could be activated by linkage through C-terminal cysteines with R_4_ or R_5_ sequences [141]. Cellular uptake in Hela cells was determined by flow cytometry for 5 µM and 20 µM concentrations. The uptake was similar to that of the control R_8_ and R_9_ peptides. Important parameters such as oligoarginine length, concentration and position of disulfide bridges (structural arrangement of arginines) were essential to improve efficiency and influence the mechanism of uptake. Symmetric distribution of guanidinium groups led to more efficient CPPs.

***Conformational restriction***. As described previously, helix stabilization can be achieved by adding alkyl chains or by including α,α-disubstituted amino acids such as Aib into poly(arginine) mimics in the primary sequence design [80,128,129]. In addition, stabilized helices can be obtained by covalent bond formation between two side chains of short peptides. Examples are disulfide bonds, stapling, and N-terminal aspartic acid crosslinking [142].

**Figure 10:**
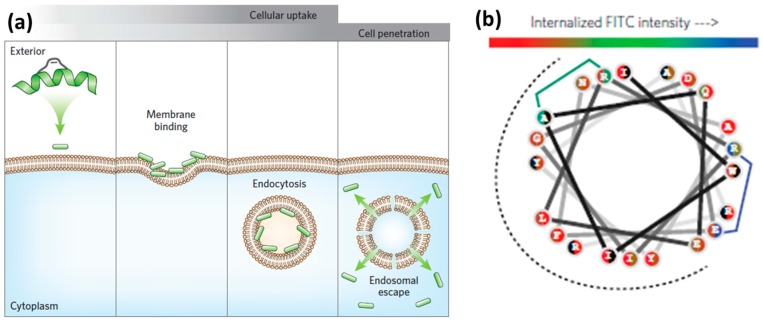
Schematic representation of (**a**) stapled CPP internalization by endocytosis followed by endosomal escape, taken with permission from reference [148], (Copyright © 2016, Nature Publishing Group) and; (**b**) wheel depiction of a stapled helix with the hydrophobic face indicated by the dotted surface and the two distinct staples indicated with blue and green lines, taken with permission from reference [146], (Copyright © 2016, Nature Publishing Group).

First described in 2000 by Verdine and colleagues, stapling consists of introducing all-hydrocarbon crosslinks into peptide sequences. This is achieved by ring-closing olefin metathesis of α,α-disubstituted non-natural amino acids that have hydrocarbon tethers [143]. Since then, peptide stapling has been successfully applied to the study of protein–protein interactions for intra- and extra-cellular targets [143,144,145]. The α-helical stabilization achieved resulted in increased resistance to proteases because the peptide bonds in the interior of the helix were protected. Staples were also explored in the CPP field as it was observed that some, but not all, α-helical staples are able to reach intracellular targets. In an attempt to unravel the biophysical properties that drive the uptake of stapled-peptide libraries, Walensky and coworkers reported on the main parameters to be included in the design of stapled CPPs (Figure 10b) [146]. Rather than the staple itself, various factors such as helical stability, increased overall hydrophobicity and positive charge were found to influence the CPP efficiency [147]. Nevertheless, the presence of the staple was found to improve hydrophobic interactions with membranes (Figure 10a). Efficient cellular uptake of the stapled peptide, when compared to the unstapled version, was shown in mouse embryonic fibroblasts (MEFs) at 500 nM dosing [146].

Recently, N-terminal crosslinking of aspartic acid has been reported to stabilize the helical structure [149]. The crosslinking was obtained by simple peptide bond formation between the side chain of the terminal aspartic acid and the amino group of the 2,3-diaminopropionic acid (Dap) to obtain a crosslink that shows a similar distance to that introduced through stapling [150]. This method was optimized for pentapeptides, and cyclization using l- and d-stereoisomers of aspartic acid was assessed. The introduction of these crosslinks was responsible for the induction or stabilization of helical conformations. This example is further evidence that the introduction of conformational constraints can improve cell permeability. This concept was taken further to study the influence of single amino acid contributions in stabilized helices. Amphipathic helical peptides containing Trp, Phe and Arg promoted cell penetration in MCF-7 and HEK293T cells when compared to those containing Lys, His, Tyr and Leu [149].

### 4.4. Controlled Self-Assembly

The early 1990s saw the emergence of a new field of molecular self-assembly based on synthetic peptides. In this regard, pioneering work was presented by Ghadiri [151] and Zhang [152]. The supramolecular assembly of molecular building blocks composed of specific peptide chains is based on the same principles as those found in nature during protein folding. The 20 gene-encoded amino acids, together with a variety of non-natural ones, provide a versatile toolbox. Today, we know that synthetic peptides can self-assemble into a variety of nanoscale morphologies, which are directed by the amino acid sequence [153] and the mode of assembly [154]. 

The process of molecular self-assembly is based on weak non-covalent interactions such as hydrogen bonds, electrostatic interactions that involve charged groups, van der Waals interactions, hydrophobic interactions, and aromatic π-stacking. Nanostructures can form as a result of interactions between complementary peptide chains that mimic the basic conformational units found in proteins. A rich chemical and structural diversity can be achieved by designing building blocks composed of sequences of amino acids (peptides) that can spontaneously assemble into nanostructures with distinct architectures. This field has experienced rapid growth, and peptide self-assembly is now increasingly studied for a wide range of applications in biomedicine, including drug-delivery, diagnostics, tissue engineering, and regenerative medicine [109,110,115,151,155,156]. Although peptide self-assembly has been exploited for several applications, only recently has this concept been introduced into the field of CPPs [36,157].

The concept that supramolecular structures can show different biological behavior to that of the monomers they are made of was introduced by Stupp and co-workers [158]. Moreover, a number of supramolecular morphologies show the ability to instruct cell death or survival by exerting a range of effects on cells. More in detail, unlike their soluble unassembled counterparts, supramolecular materials based on peptide amphiphiles with hydrophobic domains and cationic charge interact with cells. Supramolecular materials with nearly identical chemical composition can instruct cell death or survival depending on their β-sheet-forming capacity. Fragments that show better propensity to form β-sheets assemble into more cohesive fibers, where the structures reinforced by hydrogen bonding support cell viability. On the other hand, nanostructures based on weaker interactions can promote the disruption of lipid membranes and thus cause cell death. The authors reported a systematic study of the effect of hydrophobic collapse, charge and hydrogen bonding on self-assembling morphology of the peptide and cell viability. Hydrophobicity and cationic charge were found to be essential for cytotoxicity, as reducing the hydrophobic tail or the overall cationic charge resulted in lower toxicity of the peptide amphiphiles examined.

**Figure 11:**
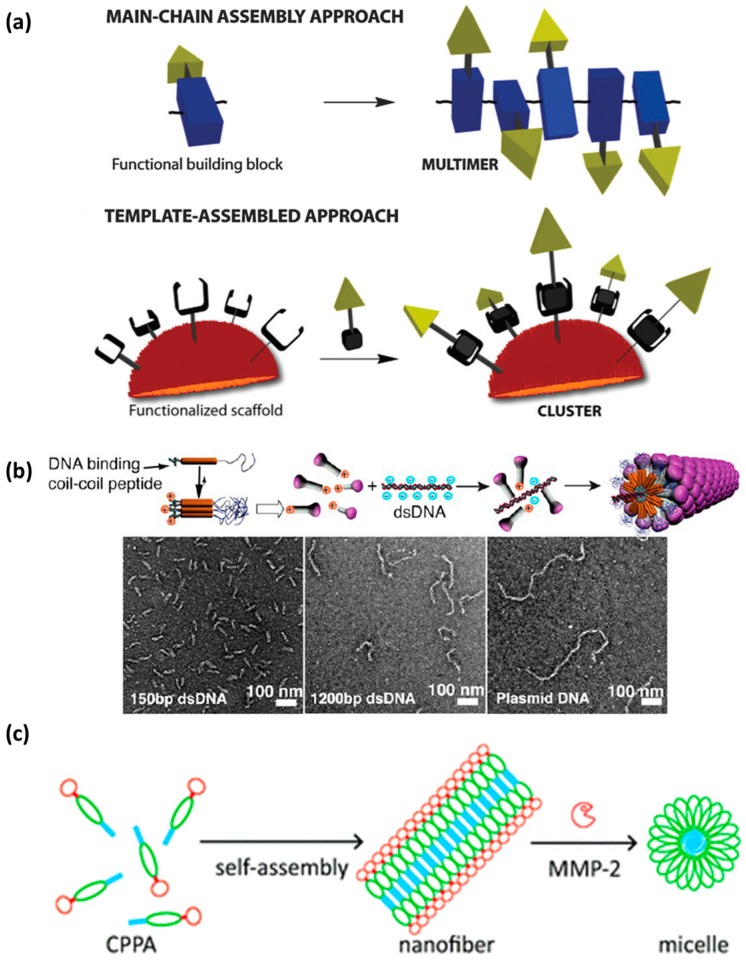
(**a**) Schematic representation of various approaches (main-chain and templated) to obtain multivalency in synthetic self-assembled systems, taken with permission from reference [132], (Copyright © 2016, Royal Society of Chemistry); (**b**) Schematic representation and TEM images of DNA-templated, supramolecular DNA-peptide 1-dimensional assemblies, with precise control over dimensions, reprinted with permission from reference [157], (Copyright © 2013, American Chemical Society); (**c**) Supramolecular, enzyme-responsive CPP amphiphiles that undergo fiber-to-micelle transition in response to MMP-2 and consequently enter cells as a result of exposure of the CPP moieties, taken with permission from reference [43], (Copyright © 2017, Royal Society of Chemistry).

In this section, examples of supramolecular CPPs based on intermolecular interaction of repeating units will be presented. Supramolecular assemblies can offer some advantages over primary and secondary CPPs. In this regard, these assembles have tunable physical properties, higher stability, multivalency, and tunable surface functionality, i.e., high local concentration of positive charge and/or hydrophobicity. In self-assembled systems, multivalency can be achieved through two main approaches: main chain or templated, as described in the review by Ulrich and co-workers (Figure 11a) [132]. Stupp and co-workers achieved precise control of the dimensions of virus-like nanostructures through molecular self-assembly [157]. Using DNA as a template, one-dimensional supramolecular structures of precise length and with tunable characteristics can be obtained (Figure 11b). These nanostructures can carry a range of cargos and show great potential for achieving cell permeability through precise design [157].

Dong and coworkers introduced the concept of filamentous CPPs (FCPPs) based on cationic multi-domain peptides, providing examples of supramolecular structure-dependent membrane activity [36,159]. The supramolecular approach to obtain FCPPs (based on β-sheet structures) was reported for cationic peptides with generic structure K_x_(QL)_y_K_xz_ or K_x_(QW)_y_K_xz_. In this case, supramolecular structures form due to the interplay of electrostatic and hydrophobic interactions alongside hydrogen bonding among QL/QW repeating units. The supramolecular assembly of these short cationic peptides into nanostructures with diverse architectures has the advantage of providing an enhanced local charge density. Supramolecular assembly resulted in dense packing of lysine residues, giving rise to high local concentrations of positive charge, i.e., multivalent species able to interact with the cell membrane. This enhanced interaction is beneficial for tuning the membrane activity of FCPPs through programmable physical properties, stability and surface functionality. The design was based on combining tryptophan and lysine at the primary structure level to obtain self-assembly into a variety of nanostructures. Tryptophan was chosen as the hydrophobic aromatic residue through which to increase contacts with membranes but also to drive the assembly. On the other hand, lysine was chosen because of positive charge, thus conferring cell penetration and serving as a DNA complexation moiety. Two molecules with structures K_5_(QW)_6_K_5_ and K_10_(QW)_6_ were compared in that study. A total of ten lysine residues were used but they were distributed in a different manner along the sequence. The molecule with a single charged domain (ten lysine residues) at the N-terminus assembled into nanofibrils based on β-sheets, while the other peptide showed random coils and irregular aggregates. The fibrillar K_10_(QW)_6_ showed greater capacity to enter Hela cells. DNA delivery with this peptide was 200- and 400-fold higher in the HEK293 and HeLa cell lines, respectively. In this example, the fibrilllar shape of the supramolecular nanostructure with the precise balance of charge distribution and conformational flexibility, was responsible for higher uptake [36]. This system was also used to deliver impermeable anticancer drugs. Similar to DNA delivery, the fibrillar nanostructures promoted higher and faster uptake of doxorubicinin into HeLa cells. Membrane sensitization was observed, as was increased permeability to doxorubicin caused by supramolecular structure and charge multivalency [159].

Tryptophan-rich CPPs that assemble into spherical aggregates were reported [160]. Their design comprised linear, tripodal and dendrimeric tryptophan-rich structures, to promote cell penetration and drive self-assembly. The dendrimer structure was based on branched lysines and contained one, three or five tryptophan residues at different geometric locations. Flow cytometry and confocal microscopy confirmed that the linear version showed the best performance and was thus a candidate for further investigation for the delivery of hydrophobic drugs [160].

Another example worthy of mention comprises triple helical CPPs obtained by combining short oligoarginine CPPs (R_6_ or RRGRRG) with collagen-like folding domains [14]. In this case, the triple helical conformation was obtained by using (proline-hydroxyproline-glycine)_n_ repeating units, which are found in collagen. The exterior of the helices was decorated with the CPPs, a process that led to the accumulation of positive charges, which favored internalization while the helicity improved resistance to proteases. Internalization efficiency (at a concentration below the 10 μM) was shown in Jurkat human leukemia cells using flow cytometry, with the R_6-_containing helix showing the highest uptake. No uptake was observed when a scrambled version of the helical motif, which was unable to assume the triple helical conformation, was used [14].

Recently, Azevedo and co-workers reported on a CPP amphiphile (CPPA) system based on the self-assembly of multidomain peptide sequences. The design consisted of four main structural and functional parameters. A hydrophobic palmitic acid (C_16_) tail that drives the self-assembly into nanofibers and serves as hydrophobic pocket for hydrophobic drug encapsulation was added at the N-terminus. Next, a CPP moiety (YTA4, sequence: IAWVKAFIRKLRKGPLG), an enzyme-sensitive moiety and an RGD-based targeting moiety were included. The system was found to self-assemble into fibers that are enzyme-responsive and that protect the CPP sequence. It was observed that RGD drives the accumulation of these fibers in the proximity of the tumor, where they can undergo enzyme-triggered fiber to micelle transition and consequently enter cells on demand as a result of exposure of their CPP moiety (Figure 11c). The in vitro evaluation of these nanostructures is needed to compare their performance in living cells.

An interesting example of quaternary structure and an alternative approach to control the uptake of oligoarginine-based CPPs was through the formation of host–guest supramolecular complexes based on anion recognition [161]. In this system, the R_8_ CPP was conjugated at the N-terminus to a negatively charged pyranine-oligoglutamic anion (guest) that was encapsulated by the positively charged polyamine cage (host). The host–guest supramolecular complexes were internalized into Vero cells and showed endosomal localization. This is an example of cell-penetrating systems in which structure and function complexity are achieved through simple design choices [161]. In addition, the cage and the peptide can be covalently modified, thereby opening up the possibility to attach a range of drugs.

Finally, the recent report from Garcia-Lopez et al. on the design of photo-activatable molecular machines able to drill through cell membranes illustrates the important role that molecular motors will have in the future in the field of intracellular delivery [162]. 

## 5. Challenges and Future Perspectives

Despite the variety of CPP design approaches currently available, the translation of CPPs to a clinical setting remains a challenge. From the examples discussed in the text, it can be appreciated that the CPP field is wide and in continuous development (Table 1). Greater mechanistic understanding and further development, often with the focus on endosomal escape enhancement, have led to an increase in design complexity. This includes the development of stable and often multi-domain cyclic or self-assembled nanostructures. Moreover, selectivity, targeting and high efficiency and environment responsiveness are sought to obtain programmable, tailored and on-demand systems.

Many researchers have proposed design strategies that allow control over folding, cyclization, dimerization, stapling, self-assembly and even CPP mimetics with different backbone structures and properties, thus leading to improvements in internalization efficiency and stability. However, due to the concomitant involvement of various internalization mechanisms, the heterogeneity of diseases, and the variety of cell lines available for in vitro studies, there are still no standards through which to rate the internalization efficiency improvements of one design over another. In addition, the heterogeneity of cargoes implies a broadening of the parameters to consider when reporting on CPP efficiency and activity. 

In this regard, standardized protocols are needed as well as a greater number of comparative studies between different classes of newly designed CPPs. Although huge progress has been made in this field, there is still room for the development of new strategies and approaches.

## Figures and Tables

**Table 1:** Examples of CPPs and their sequences, α-carbon stereochemistry, source, design strategy, structural features and development stage.

Entry	CPP Name	Sequence	α-Carbon Configuration	Source	Design Approach	Structural Features	Development Stage	Ref.
Linear Peptides								
**1**	Tat	GRKKRRQRRRPQ	*all*-l	Natural (transcription protein of HIV-1-positions 48–60)	Virus derived material	Unstructured in buffer solutions; random coil	In clinical trials	[2,3,8,9,15]
**2**	Penetratin	RQIKIWFQNRRMKWKK	*all*-l	Natural (Drosophila Antennapedia homeodomain)	Derived from natural Antennapedia homeoprotein	Secondary amphipathic; forms helices or β-sheets depending on the environment	In vivo data	[4,9,15,76,120,163,164]
**3**	Pep-1	KETWWETWWTEWSQP-KKKRKV	*all*-l	Fusion CPP: Trp-rich segment; Lys-rich segment; and NLS derived from a virus (SV-40 T-antigen)	Combination of designed and natural segments	Primary amphipathic; forms helices in the presence of phospholipids	In vivo data	[8,9,15,22]
**4**	MPG	GALFLGFLGAAGSTMGAWSQP-KKKRKV	*all*-l	Fusion CPP: NLS derived from a virus (SV-40 T-antigen) and viral hydrophobic domain from HIV-gp-41 segment	Combination of natural segments	Primary amphipathic; forms helices in the presence of phospholipids	In vivo data	[8,23]
**5**	Polyarginine (R_9_, R_8_)	RRRRRRRRR	*all*-l	Design inspired by entry 1 and 2	Designed to be R-rich	Flexible; unstructured; random coil	In clinical trials	[8,9,15,18,19,20,34,56]
**6**	R_6_/W_3_	RRWWRRWRR	*all*-l	Design inspired by entry 2	Designed to be R-rich and hydrophobic	Secondary amphipathic, forms helices in the presence of phospholipids	Cell assays	[9,15,165]
**7**	SAP	(VRLPPP)_3_ (vrlppp)_3_	*all*-l *all*-d	Design inspired by a natural protein of maize, γ-zein VHL(PPP)_8_	Derived from natural γ-zein protein	Polyproline II helical structure	Cell assays; In vivo data	[24,39,79,103,122,123]
**8**	SAP(E)	Ac-CGGW(VELPPP)_3_	*all*-l	Design inspired by SAP; Arg residue replaced by Glu	Designed to be negatively charged	Polyproline II helical structure	Biophysical data	[124,125]
**9**	CyLoP-1	CRWRWKCCKK	*all*-l	Derived from crotamine toxin found in snake venom, crot(27–39)	Rationally designed, by substitution/ deletion of crot(27–39) sequence	Disulfide-containing form effective; oxidation status of the cysteines important for the uptake	Cell assays	[25]
**10**	gH 625	HGLASTLTRWAHYNALIRAF	*all*-l	Natural; based on the 625–644 residues of the glycoprotein HSV 1	Derived from Herpes Simplex virus type I (HSV 1)	Amphipathic; α-helical conformation in contact with model membranes	In vivo data	[26,27,28,29,30,32,33,166]
**11**	GALA	WEAALAEALAEALAEHLAEALAEALEALAA	*all*-l	Glu-rich an containing His (imidazole group) in order to be pH responsive (endosomes)	Designed to efficiently escape endosomes	Flexible but assumes helical structure able to lyse endosomal membranes	In vivo data	[55,67,68]
**12**	TP10	AGYLLGKINLKALAALAKKIL	*all*-l	Fusion CPP: N-terminal amino acids from galanin (AGYLLGKINLK) linked to matoparan (ALAALAKKIL)	Derived from the neuropeptide galanin linked to a toxin from the wasp venom	Primary amphipathic; forms helices in the presence of phospholipids	In vivo data	[8,9,167]
**13**	CADY	Ac-GLWRALWRLLRSLWRLLWRA-cysteamide	*all*-l	Designed; based on chimeric peptide carrier PPTG1 derived from the fusion peptide JTS1	Designed by combining aromatic (W) and cationic (R) residues into a secondary amphipathic CPP	Secondary amphipathic; helical conformation	Cell assays	[5,102,119]
**14**	L17E	IWLTALKFLGKHAAKHEAKQQLSKL	*all*-l	Natural; inspired by the spider venom M-lycotoxin	Designed to contain E residues in the hydrophobic part of the amphipathic helix	Secondary amphipathic	Cell assays	[69]
**15**	MPPs	Mitochondria-penetrating peptides (example: F_X_rF_X_KF_X_rF_X_K)	Combination of l- and d-	Designed to contain un-natural, cyclohexylalanine (F_x_) residues	Designed to have differential intracellular localization	Flexible	In vivo data	[91,92]
**16**	Ac-1 * Ent Ac-1 Ac-2	Ac-(RR-Aib)_3_ Ac-(rr-Aib)_3_ Ac-(Rr-Aib)_3_	*all*-l, *all*-d or combination of l- and d-Arg	Designed to contain Aib, natural non-coded amino acid	Helix stabilization is introduced at the primary sequence level	α-Helical structure	Cell assays	[129]
**17**	Peptide 3	FAM-β-Ala-(RRP^Gu^)_3_G_3_	*all*-l	Designed to contain -L proline or guanidinyl -L proline	Pro residue introduced in order to sense hydrophobic and amphipathic environments	Helical structure in contact with membranes	Cell assays	[130]
**18**	RR5-App RR4-App * RR3-aPP	RRPRRPRRPRRPGRR-APVEDLIRFYNDLQQYLNVVTRHRYC RRPRRPRRPGRR-APVEDLIRFYNDLQQYLNVVTRHRYC GPRRPRRPGRR-APVEDLIRFYNDLQQYLNVVTRHRYC	*all*-l	Small proteins (36-residue polypeptides)	Arg residues were located on the solvent-exposed side of PPII helices	PPII-type helix	Cell assays	[47,131]
**19**	TATp-D	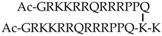	*all*-l	Designed as analogue of Tat	Covalent dimeric branched peptide; dimerization obtained through bis-Fmoc protected lysine near the C-terminus	Branched peptide	Cell assays	[135]
**20**	R4–R4 * R5–R5	RRRRC-CRRRR RRRRRC-CRRRRR	*all*-l	Extended peptides obtained through disulfide bridge formation of truncated oligoarginines	Designed as small inactive oligoarginine fragments (R_4_) activated by linkage through C-terminal cysteines	Branched peptide	Cell assays	[141]
Cyclic Peptides								
**21**	[WR]_4_ * ([FK]_4_, [AK]_4_, [EL]_4_, [RFEF]_2_, [EK]_4_, [ER]_4_, [FR]_4_, [RFE]_3_, [WR]_3_, [WR]_5_)	c[WRWRWRWR]c	*all*-l	Designed to contain combinations of hydrophobic (W, F, L) and charged (R, K, E) residues	Designed to obtain optimal amphipathic CPP resistant to proteolysis	Cyclic	Cell assays	[136]
**22**	Cyclic Tat	c[K-rRrQrRkKrG-E]c	Combination of l- and d-	Lys- and Glu- amino acids added to the linear Tat sequence to obtain a ring with the same overall charge as the native form	Designed to introduce structural rigidity and controlled spatial distribution of guanidinium groups	Cyclic	Cell assays	[21]
**23**	cFΦR_4_	c[FΦRRRRQ]c	*all*-l	Designed to contain Φ (L-2-naphthylalanine)	Varying design parameters used: sequence lengths; stereochemistry; or combination of the two	Cyclic	In vivo data	[60,133]
**24**	Danamide D	c-[I(Thz)-tBuGly-FPIP]	*all*-l	Design based on cyclic heptapeptide sanguinamide A	Rigid scaffold obtained through formation of a heterocycle; rigidity reinforced through bulky hydrophobic tertbutyl glycine side chains	Cyclic	In vivo data	[105]
**25**	Pro-(Xaa)_4_-Tyr	c[d-Pro−l-MeLeu−d-MeLeu−d/l-MeLeu−d/l-Leu−l-Tyr]c *	*all*-l, *all*-d or combination of l- and d-	Inspired by cyclic natural products Guangomide A and baceridin	Stereochemistry and N-methylation used to obtain backbone geometries with different conformational preferences	Cyclic	Biophysical data	[137]
**26**	Cyclic sC18	c[GLRKRLRKFRNK]c-IKEK *	*all*-l	Inspired by the CAP-18 antimicrobial peptide; cyclization obtained by connecting residues at positions 1 + 4, 1 + 8 and 1 + 12 of the linear peptide	Gly and Lys residues replaced by propargylglycine and ε-azidolysine to allow cyclization through triazole bridge formation by click chemistry	Cyclic	Cell assays	[49]
**27**	RRRRΦF *	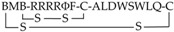	*all*-l	Bicyclic compounds; cyclization through cysteine side chains and 3,5-bis(mercaptomethyl) benzoic acid (BMB)	Designed to be conformationally constrained with the goal to induce the uptake of generally impermeable peptidyl drugs	Cyclic	Cell assays	[134,138]
**28**	BIM SAHB9 SAH–SOS1	IWIAQELRXIGDXFNAYYARR * ZFGIYLTXILKTEEGN	*all*-l	Designed to have i + 4 staples (between X pairs; X is S-pentenylalanine) or i + 7 staples (between Z and X; Z is R-octenyl alanine	Hydrophobic staple introduced for α-helical stabilization	Stapled	Cell assays	[146]
**29**	4-R 4-W	FITC-β-A (iso-DRRX)WRRW FITC-β-A (iso-DWWX)RWWR	l- or d-stereoisomers of Asp used	Peptide bond formation between the side chain of the terminal Asp and the -NH_2_ of Dap (X is Dap) to obtain a crosslink	Introduction of crosslinks (at similar distance to that introduced through stapling) to stabilize helical conformations	N-terminal crosslinking	Cell assays	[149]
Supramolecular CPPs								
**30**	Sp-CC-PEG_2000_	Sp-REGVAKALRAVANALHYNASALEEVADALQKVKM-PEG	*all*-l	Obtained by the self-assembly of coiled-coil peptides decorated with cationic segments (Sp = spermine) and PEG placed at opposite termini	Supramolecular structures form due to the interaction of the peptide derivative with a DNA segment	Supramolecular filamentous virus-like nanostructures	Biophysical data	[157]
**31**	K_10_(QW)_6_	KKKKKKKKKKQWQWQWQWQWQW	*all*-l	The design was based on combining W and K at the primary structure level to obtain self-assembly into a variety of nanostructures	Supramolecular structures form due to the interplay of electrostatic and hydrophobic interactions alongside with hydrogen bonding among QL/QW repeating units	Supramolecular fibers (based on β-sheets)	Cell assays	[36,159]
**32**	YTA4	C_16_-IAWVKAFIRKLRKGPLG-GPLGIAGQ-RGDS	*all*-l	CPP amphiphile system based on the self-assembly of multi-domain peptide sequences	Design based on four main structural and functional parameters: palmitic acid (C_16_) tail to drive self-assembly into nanofibers; a CPP moiety; an enzyme-sensitive moiety; and an RGD-based targeting moiety	Self-assembly into nanofibers	Biophysical data	[43]
**33**	V2	FITC–AβGG-POGPOGPOGPOGPOGPOGPOGPOGRRRRRR	*all*-l	Designed to contain (POG)_n_ collagen like motif to induce triple helix formation (O is hydroxyproline)	Stable helix obtained inspired by collagen decorated with R_6_	Triple helix	Cell assays	[14]
**34**	W_3_	WWW	*all*-l	The design comprised linear, tripodal and dendrimeric W rich structures	Design based on self-assembly info spherical aggregates	Spherical aggregates	Cell assays	[160]
Peptido mimetics								
**35**	CPPMs	Synthetic mimics of CPPs	Polypeptides obtained by ring opening polymerization	Designed to mimic Tat	Designed to have a polymeric backbone with guanidines at specific positions, aromatic or aliphatic functionality	Flexible	Biophysical data	[81,88,97,98]
**36**	Peptoids (CPPos)	Peptidomimetic molecules	Not applicable	Have side chains on the nitrogen atom rather than on α-carbon	Specific organelle localization	Flexible	Cell assays	[89,90]
**37**	CPDs	Cell-penetrating disulfides	Not applicable	Designed to have a polydisulfide backbone; guanidinium rich	Designed to be responsive to reductive environments	Flexible/disulfide bridges	Cell assays	[13,93,94,95,96]

* Several related sequences reported; only a representative example shown in the table.
